# Psychological effects of, and compliance with, self-isolation among COVID-19 patients in South Batinah Governorate, Oman: a cross-sectional study

**DOI:** 10.1186/s41983-022-00481-x

**Published:** 2022-05-08

**Authors:** Zayid K. Almayahi, Nasser Al Lamki

**Affiliations:** Disease Surveillance and Control Department, Ministry of Health, South Batinah Governorate Rustaq, Sultanate of Oman

**Keywords:** Oman, Stress, Psychology, Compliance, COVID-19, Prevalence, Quarantine

## Abstract

**Background:**

Covid-19 pandemic has left deep psychological impacts, especially among infected patients. It is extremely important to understand the extent of those effects, while improving the compliance with isolation measures at the same time.

**Objectives:**

To detect prevalence of stress using two psychological scales and examine the stress associated factors, also to identify self-isolation compliance rates among COVID-19 patients.

**Methods:**

Cross-sectional research was conducted from 15 November to 22 December 2020, involving 379 patient participants selected via systematic random sampling. Kessler 10 Psychological Distress (K10) and the impact of event scale-revised (IES-R) tests were used to ascertain the levels of distress.

**Results:**

K10 measure revealed elevated stress amongst 121 (31.9%) of participants, whereas IES_R indicated the level was 37.7%. Using the K10 indicated the multivariate analysis was significant for females (OR = 2.482, 95% CI: 1.532–4.021), patients with financial problems (OR = 2.332, 95% CI: 1.270–4.282) and patients experiencing shortages of essentials (OR = 4.920, 95% CI: 2.524–9.590). The IES-R scale indicated that only female and patients experiencing shortages scored significantly in multivariate analysis, (OR = 1.895, 95% CI: 1.1223–2.935) and (OR = 2.928, 95% CI: 1.1580–5.424), respectively. Those undergoing shorter isolation periods reported lower levels of stress on both K10, p=0.016 and IES-R, p=0.002. Approximately 90% of patients used their own towels during isolation. Moreover, 80.2% slept in separate rooms and 74% used masks in the presence of other family members. Essential supply shortages were reported by 14.2% of respondents.

**Conclusions:**

Self-compliance rates were not optimal, while psychological distress was more prevalent among some groups. Intervention is imperative to minimize stress and improve self-isolation compliance.

## Introduction

The COVID-19 pandemic was unforeseen both in its occurrence and its duration. Moreover, its impacts have been multiple and wide-ranging, encompassing many areas of life, including mental health [1, 2].

By 16 July 2021, there had been over 188 million cases reported globally, with the number of deaths exceeding four million [3]. Various negative psychological outcomes have been observed during both the current and previous pandemics and epidemics. For example, during the 2003 Severe Acute Respiratory Syndrome (SARS) outbreak in Canada, the psychological distress experienced by health care workers was significantly higher than was the case amongst the general population [4]. During the recent Middle East Respiratory Syndrome Coronavirus (MERS-CoV) outbreak in Korea in 2015, stress levels were not only higher, but also were reported to have persisted for longer, especially among medical staff [5]. As might be expected, the COVID-19 pandemic is no exception in that it has significantly impacted mental and psychological wellbeing, especially among high-risk groups, those already infected, or those subjected to quarantine [6–8].

However, it is also essential to comprehend the adherence to the quarantine measures demonstrated by the wider populace, not least those diagnosed with COVID-19. Different experiences across several countries have located variations in rates of compliance during the current pandemic [9]. Although many studies have explored the mental health aspects of pandemic-related confinement across the world, few have focused specifically on compliance rates during self-isolation amongst patients infected with COVID-19.

The current study comprises the first research based in Oman that has sought to determine self-quarantine compliance rates amongst COVID-19 patients. Moreover, this study employs the Kessler 10 Psychological Distress (K10) test and the impact of event scale-revised (IES-R) test to ascertain levels of distress and to examine related variables.

## Methods

### Setting

The South Batinah Governorate (SBG) is located in the north of Oman and has a population of 465,550 [10]. It is divided into six states, namely: Barka, Rustaq, Musanaa, Nakhal, Wadi Mawel and Awabi, in order of population size. This study was conducted as a cross-section between 15 November 2020 and 22 December 2020. The inclusion criteria required patient participants to belong to SBG, to have had a verified diagnosis of COVID-19 through a Polymerase Chain Reaction (PCR) test prior to 6 November 2020, to be aged 18 years or over and to be listed in the SBG disease surveillance database. Cases which failed to satisfy these criteria were excluded from the study. A small number of patients were found to belong to SBG, but were staying outside during isolation period were also included. A complete list of all patients was provided by the Department of Disease Surveillance and Control, along with their mobile contact numbers.

### Sampling

The total number of confirmed COVID-19 cases in SBG was 12,108 as of 7 November 2020. However, after excluding patients under 18 years and those who belong to other governorates within Oman, the number was reduced to 11,223 patients. These patients were listed within the database in ascending order in accordance with the date of their confirmed diagnosis. Epi Info software (version 7.2.2.6; Centers for Disease Control and Prevention (CDC), Atlanta, Georgia USA) was employed to estimate the sample size. Thus, based on the assumption that 50% of participants were aware and compliant with health isolation measures and experienced moderate levels of stress, with a 95% confidence interval and a design effect of one, the ultimate sample size was 371. However, the sample size was increased to compensate for possible losses, wrong contact numbers and patients refusing to participate. Using a systematic random sampling (*k* = 28) and a random start for selection of the first participant, 400 participants were selected. Whenever participants either could not be reached via their mobile numbers or they declined to participate, the next patient on the list was contacted as an alternative. In total, 379 participants completed the full questionnaire and were included in the analysis.

### Instruments

A predesigned questionnaire was created using Microsoft Forms and distributed to all participants via a WhatsApp link. It was bilingual (Arabic and English), thereby allowing participants to select the language they preferred to use. In addition, the questionnaire was designed to be compatible with smart phones, laptops, desktops and tablets.

Before the questionnaire was distributed to the mobile phones of participants, they received a phone call from two trained personnel, the objective of the conversation being not only to outline the research objectives and content, but also to obtain verbal consent.

The questionnaire included four major components, the first of which was a set of sociodemographic questions designed to acquire data pertaining to nationality, gender, education, residence, work, income, medical history and social status.

The second set of questions explored the conditions of health isolation, including duration, place, conception, medical service, challenges and compliance with isolation protocols.

Thirdly, there were questions designed to elicit information about the psychological stress levels associated with health isolation, which were measured using two validated scales, to wit: K10 and IES-R, both of which were available in validated English and Arabic language versions. The K10 is an attractive and simple tool, with strong psychometric properties, wherein psychological distress can be assessed through 10 questions. These questions evaluate the frequency of different symptoms experienced in the preceding 4 weeks on a scale of 1–5, where 1 = none at all and 5 = all the time. Hence, the total results range from 10 to 50 [11, 12]. The IES-R is an appropriate instrument for evaluating the subjective distress resulting from a traumatic life event. This instrument assesses symptom frequency for 22 items on a five-point Likert scale, where 0 = Not at all and 4 = extremely. The results range from 0 to 88. The IES-R has three subscale domains (avoidance, intrusion and hyperarousal) where the calculated mean provides insights into the level of distress experienced [13, 14].

The fourth question set comprised three questions designed to obtain self-evaluations of medical services using a five-point Likert scale, wherein 1 denoted “very poor”, 2 signified “poor”, 3 represented “fair”, 4 equated to “good” and 5 comprised “excellent”.

The Ministry of Health (MOH) protocol required all individuals who had been in contact with confirmed cases to quarantine for 14 days. Any individual with a positive COVID test was obliged to self-isolate for a minimum of 10 days from the test result date onwards. Isolation ceased after 10 days, provided the individual had been symptom-free for the previous 2 days [15].

Whilst some individuals were obliged to isolate for less than 14 days, other individuals may have been in isolation for longer periods. Such cases included those who had already been in isolation for several days prior to a positive test result and individuals who exhibited long-lasting symptoms.

Participants were classified into two groups, based on the month of diagnosis, because the first few months of March through June were epidemiologically classed as cluster transmission cases in Oman, whereas cases from July to November occurred at a time when community transmission became real.

Monthly income was defined as negatively impacted when the patient or her/his spouse or any breadwinner was not paid for a number of weeks or months during the pandemic. This also included cases where individuals had lost their jobs during the pandemic or where there was a salary deduction related to the isolation period.

### Statistical analysis

The data collected through the Microsoft Form was exported as a Microsoft Excel file before being organised, tabulated and statistically analysed using IBM SPSS 25.0 (IBM Corp., Armonk, New York USA). Subsequently, the resultant numerical data were presented as means and standard deviations, whilst the categorical data were presented as numbers and percentages.

The reliability test was calculated using the Cronbach’s alpha (α) for both the K10 and IES-R scales; 0.893 and 0.922 respectively. Hence, the three self-evaluation questions pertaining to medical service provision and clinical, psychological and socioeconomic aspects produced a Cronbach’s alpha (α) of 0.773.

The K10 total score was divided into four sub-categories, comprising low (10–15), moderate (16–21), high (22–29) and very high (30–35) [16]. A chi-squared test was used to identify differences between the sub-categories. The binary coding for K10 was used in the logistic model, with the combination of low and moderate levels indicating low distress (scores of 21 or less), while high distress comprised the combination of high and very high (scores of 22 or greater).

The total score of IES-R was further divided into high and low stress. Scores of 25 or higher were considered high stress [17] and the mean and standard deviation (SD) were calculated for the subcategories of the independent variables. In addition, the normality assumption was evaluated via both the Kolmogorov–Smirnov test and visual assessment, whilst the Mann–Whitney *U* test and the Kruskal–Wallis test were used to compare the subgroups. Odds ratios (OR) and related 95% confidence intervals (95% CI) were calculated using bivariate and multivariable analyses (unconditional binary logistic regression). Only statistically significant covariates in the bivariate analyses were included in the multivariable model. The *p* value adopted was *p* ≤ 0.05.

### Ethical considerations

Prior to the administration of the questionnaire, verbal consent was obtained from all participants through telephone conversations with two trained team members. This was supplemented with the electronic consent provided by each respondent during the questionnaire process. Participants could opt to complete English or Arabic language questionnaires. All data was collected anonymously and only used for research purposes. Confidentiality was safeguarded throughout the research process. Ethical approval was sought and obtained on 21 July 2020 from the Research and Ethical Review and Approval Committee, Directorate Planning and Studies at the South Batinah Governorate (Research Code 02072020).

## Results

Table 1 describes the demographic characteristics of the participants. A total of 379 participants completed the questionnaires, of whom 363 (95.8%) were Omanis and 231 (60.9%) were men. The age groups ≤ 30 and 31–40 contained equal participants, comprising149 (39.3%) each. Different chronic medical problems were reported by 81 (21.4%) participants. The two-week isolation period was completed by 201 (53%) participants, as compared to the 138 (36.4%) who experienced longer isolations periods. Monthly income decreased for 66 (17.4%) participants.

**Table 1:** Participant’s characteristics

	*N* = 379	%
Nationality		
Omani	363	95.8
Non-Omani	16	4.2
Gender		
Man	231	60.9
Woman	148	39.1
Place		
Barka	144	38
Musanaah	98	25.9
Rustaq	76	20.1
Nakhal	22	5.8
Wadi Al Mawel	14	3.7
Awabi	11	2.9
Outside SBG	14	3.7
Age		
≤ 30	149	39.3
31–40	149	39.3
> 40	81	21.4
Social status		
Married	278	73.4
Single	84	22.2
Divorced and widowed	17	4.5
Educational level		
Primary school	71	18.7
Secondary school	153	40.4
Diploma/Bachelor/or higher	155	40.9
Place of work		
Governmental	162	42.7
Private	79	36.4
I don’t have work	138	20.8
Working in the health sector		
Yes	32	8.4
No	347	91.6
Number of children		
1–2 children	110	29
≥ 3 children	153	40.4
I don’t have children	116	30.6
Number of household members		
5 or less	150	39.6
6–10	162	42.7
11 or more	66	17.4
Missing	1	0.3
Comorbidities		
Yes	81	21.4
No	298	78.6
Isolation period		
14 days	201	53
Less than 14 days	40	10.6
More than 14 days	138	36.4
Month of diagnosis		
February–June	119	31.4
July–November	260	68.6
Monthly income affected		
Yes	66	17.4
No	313	82.6
Health facility communication		
No	26	6.9
Yes, daily	115	30.3
Yes, few times	238	62.8

Home-based self-isolation applied to 353 (93.1%) participants. Most participants (370 or 97.6%) appreciated that isolation was necessary to protect others. However, 5% reported they principally complied with isolation due to pressure exerted by the Ministry of Health, the police and the community. With respect to isolation behaviour, 344 (90%) participants used personal towels and 319 (84.2%) slept in separate rooms. Table 2 includes other characteristics pertaining to isolation behaviour.

**Table 2:** Characteristics of the isolation, stress scales, and self-evaluation of participants

Isolation setting and characteristics	*n*	%
Home isolation	353	93.1
Governmental, work, or separate isolation	26	6.9
Isolation conception		
I am persuaded of the value and necessity of isolation	370	97.6
I am complied under the pressure of MOH, police or community	18	4.8
Isolation behaviour		
Always sleeping in a separated room	319	84.2
Using personal towels	344	90.8
Using masks in the presence of other family members	279	73.6
Putting wastes in double bags	293	77.3
Using masks when going outdoors for necessary purposes	329	86.8
Going out during isolation period for socializing	19	5
Receiving visitors in your home	8	2.1
Going out for important visits only	81	21.4
Going out for drive	42	11.1
Taking care of children	96	25.3
Challenges and support during isolation		
Shortage in any of essential supplies of house	54	14.2
Received support of relatives or friends	204	53.8
Support by someone from the house not under quarantine	179	47.2
Shopping online or calling the nearby shops	34	8.9
I had to bring house staff myself	12	3.2
Charitable organization support	6	1.6

Psychological measures		
Kessler Score High stress	121	31.9
Low stress	258	68.1
IES_R score High	143	37.7
Low	236	62.3

	Mean	SD (CI 95%)
Kessler Score	19.47	7.996 (18.67–20.28)
Total IES_Revised	21.27	17.414 (19.52–23.03)
Avoidance subscale	7.98	7.591 (7.22–8.75)
Intrusion subscale	7.32	6.788 (6.63–8.00)
Hyperarousal subscale	5.97	4.902 (5.48–6.47)
Self-evaluation of health services		
Clinical progress	4.14	0.775 (4.06–4.23)
Psychological aspect	4.11	0.771 (4.02–4.19)
Socio-economic status	3.90	0.778 (3.82–3.99)
Overall satisfaction	7.07	2.775 (6.76–7.380

The K10 results indicated that 121 (31.9%) participants suffered from high levels of stress, as compared to the 143 (37.7%) in the IES_R score. The means and SDs for the avoidance, intrusion and hyperarousal subscales were 7.98 ± 4.902, 7.32 ± 6.788 and 5.97 ± 4.902, respectively.

Table 3 illustrates the relationship between the K10 categorical scores and participant characteristics. High stress was experienced by 23.6% of women and 16.5% of men, whilst 18.9% of women and 8.7% of men experienced very high stress, *p* = 0.001.

**Table 3:** Relationship between Kessler categorical scales and patients’ characteristics

	Low (10–15)	Moderate (16–21)	High (22–29)	Very high (30–50)	Total (%)	*X*^2^	*p* value
Nationality							
Omani	41.0	27.0	19.3	12.7	100	0.981	0.806
Non-Omani	31.3	37.5	18.8	12.5	100		
Gender							
Man	47.2	27.7	16.5	8.7	100	16.192	0.001*
Woman	30.4	27.0	23.6	18.9	100		
Place							
Awabi	54.5	27.3	18.2	-	100	9.239	0.954
Barka	38.9	27.8	18.8	14.6	100		
Musanaa	39.8	26.5	22.4	11.2	100		
Nakhal	36.4	22.7	27.3	13.6	100		
Rustaq	42.1	28.9	18.4	10.5	100		
Wadi Mawel	42.9	21.4	14.3	21.4	100		
Outside SBG	50.0	35.7	-	14.3	100		
Age							
Less than 30	40.3	26.2	21.5	12.1	100	3.245	0.778
31–40	42.3	25.5	17.4	14.8	100		
More than 40	38.3	33.3	18.5	9.9	100		
Social status							
Married	41.0	27.7	19.4	11.9	100	1.804	0.937
Single	41.7	25.0	19.0	14.3	100		
Divorced and widowed	29.4	35.3	17.6	17.6	100		
Education							
Primary	45.1	23.9	16.9	14.1	100	2.604	0.857
Secondary	40.5	25.5	20.3	13.7	100		
Diploma/Bachelor/higher	38.7	31.0	19.4	11.0	100		
Work							
Governmental work	40.7	30.9	18.5	9.9	100	12.280	0.056
Private	35.5	29.0	17.4	18.1	100		
I don’t work	49.4	17.7	24.1	8.9	100		
No of children							
1–2 Children	35.5	32.7	19.1	12.7	100	3.197	0.784
≥ children	41.2	26.8	19.0	13.1	100		
I don’t have children	44.8	23.3	19.8	12.1	100		
No of household members							
5 or less	41.3	24.0	24.0	10.7	100	5.680	0.460
6–10	38.9	31.5	16.7	13.0	100		
11 or more	43.9	25.8	15.2	15.2	100		
Comorbidities							
No	41.9	29.2	18.1	10.7	100	7.183	0.066
Yes	35.8	21.0	23.5	19.8	100		
Duration of isolation							
14 days	38.3	30.8	20.9	10.0	100	15.578	0.016*
Less than 14 days	62.5	22.5	7.5	7.5	100		
More than 14 days	37.7	23.9	20.3	18.1	100		
Communication by the health facility							
No	23.1	34.6	23.1	19.2	100	4.505	0.609
Yes, daily	44.3	27.0	18.3	10.4	100		
Few times	40.8	26.9	19.3	13.0	100		
Salary affected							
Yes	33.3	15.2	22.7	28.8	100	22.348	< 0.001*
No	42.2	30.0	18.5	9.3	100		
Month of diagnosis							
February–June	42.0	27.7	20.2	10.1	100	1.073	0.783
July–November	40.0	27.3	18.8	13.8	100		
Shortage of essential items							
No	45.5	28	17.2	9.2	100	39.89	< 0.001*
Yes	11.1	24.1	31.5	33.3	100		

*Significant result *p* ≤ 0.05

Participants who isolated for over 14 days experienced more stress (18.1%) as compared to those who spent exactly 14 days (10%), *p* = 0.016. Those whose incomes had fallen reported more stress (28.8%), *p* ≤ 0.001. Those who reported supply shortages suffered more stress (33.3%) as compared to those who had no shortages (9.2%), *p* ≤ 0.001. The multivariate analysis of the association with high stress using K10 was significant for women (OR = 2.482, 95% CI: 1.532–4.021), patients with financial problems (OR = 2.332, 95% CI: 1.270–4.282) and those who lacked essential supplies (OR = 4.920, 95% CI: 2.524–9.590), as indicated in Table 4.

**Table 4:** The factors associated with high stress (Kessler score): binary logistic regression

	Unadjusted OR				Adjusted OR			
	OR	95% CI		*p* value	OR	95% CI		*p* value
Nationality								
Omani	1.00	–	–	–				
Non-Omani	0.968	0.329	2.850	0.953				
Gender								
Man	1.00	–	–	–	1.00	–	–	–
Woman	2.211	1.422	3.437	< 0.001	2.482	1.532	4.021	< 0.001
Place								
Rustaq	2.444	0.505	11.831	0.267				
Musanaah	3.046	0.644	14.416	0.160				
Barka	3.000	0.645	13.945	0.161				
Awabi	1.333	0.157	11.356	0.792				
Wadi Mawel	3.333	0.522	21.277	0.203				
Nakhal	4.15	0.743	23.229	0.105				
Outside SBG	1.00	–	–	–				
Age								
Less than 30	1.00	–	–	–				
31–40	0.941	0.580	1.526	0.805				
More than 40	0.785	0.435	1.417	0.422				
Social status								
Married	0.835	0.299	2.331	0.731				
Single	0.917	0.307	2.735	0.876				
Divorced and widowed	1.00	–	–	–				
Education								
Primary school	1.00	–	–	–				
Secondary school	1.147	0.627	2.098	0.657				
Diploma/bachelor or higher	0.969	0.527	1.781	0.920				
Work								
Government	0.808	0.452	1.444	0.472				
Private	1.122	0.625	2.014	0.699				
I don’t work	1.00	–	–	–				
No of children								
1–2 children	0.996	0.569	1.744	0.990				
More than 3	1.006	0.600	1.688	1.006				
No children	1.00	–	–	–				
No of household members								
5 or less	1.220	0.654	2.276	0.531				
6–10	0.968	0.519	1.807	0.968				
11 or more	1.00	–	–	–				
Comorbidities								
No	1.00				1.00	–	–	–
Yes	1.876	1.131	3.111	0.015	1.603	0.918	2.797	0.097
Duration of isolation								
14 days	1.00	–	–	–	1.00	–	–	–
Less than 14	0.396	0.158	0.991	0.048	0.88	0.145	1.034	0.058
More than 14	1.398	0.887	2.204	0.149	1.208	0.735	0.985	0.456
Regular communication								
No	1.533	0.673	3.496	0.309				
Daily	0.841	0.517	1.369	0.487				
Few times	1.00							
Salary affected								
No	1.00	–	–	–	1.00	–	–	–
Yes	2.760	1.605	4.748	< 0.001	2.332	1.270	4.282	0.006
Month of diagnosis								
February–June	1.00	–	–	–				
July–November	1.120	0.700	1.790	0.636				
Shortage essential items								
No	1.00	–	–	–	1.00	–	–	–
Yes	5.119	2.78	9.426	< 0.001	4.920	2.524	9.590	< 0.001

The IER-S revealed similar results to K10 in terms of their association with gender, the duration of isolation, salary impact and supply shortages as demonstrated in Table 5. However, the multivariate analysis was significant only for females (OR = 1.895, 95% CI: 1.1223–2.935) and supply shortages (OR = 2.928, 95% CI: 1.1580–5.424) (see Table 6).

**Table 5:** Relationship between IES-R means and patients’ characteristics

	Mean	SD	*p* value
Nationality^†^			
Omani	21.53	17.59	0.229
Non-Omani	15.44	11.90	
Gender^†^			
Man	19.06	16.50	0.003*
Woman	24.73	18.28	
Place^‡^			
Awabi	22.09	22.59	0.957
Barka	21.56	18.05	
Musanaa	20.57	16.85	
Nakhal	24.05	18.08	
Rustaq	21.11	15.68	
Wadi Mawel	21.14	21.59	
Outside SBG	19.36	16.94	
Age^‡^			
Less than 30	21.01	17.80	0.141
31–40	20.66	17.01	
More than 40	22.90	1756	
Social status^‡^			
Married	20.85	16.77	0.671
Single	21.56	18.28	
Divorced and widowed	26.88	22.90	
Education^‡^			
Primary	22.72	19.02	0.782
Secondary	21.16	16.92	
Diploma/Bachelor/ or higher	20.73	17.21	
Work^‡^			
Governmental work	20.16	16.63	0.525
Private	22.96	18.58	
I don’t work	20.62	16.86	
No of children‡			
1–2 Children	20.64	16.79	0.653
≥ 3 children	22.22	17.70	
I don’t have children	20.64	17.71	
No of household members^‡^			
5 or less	20.81	17.84	0.788
6–10	21.49	17.84	
11 or more	21.23	17.14	
Comorbidities†			
No	20.55	16.68	0.328
Yes	23.95	19.78	
Duration of isolation^‡^			
14 days	20.63	16.60	0.002*
Less than 14 days	13.37	10.34	
More than 14 days	24.50	19.36	
Communication by the health facility^‡^			
No	26.12	20.57	0.498
Yes, daily	20.01	16.90	
Few times	21.36	17.274	
Salary affected^†^			
Yes	27.79	19.91	0.003*
No	19.90	16.55	
Month of diagnosis^†^			
February–June	20.63	16.60	0.876
July–November	21.57	17.80	
Shortage of essential items			
No	19.35	16.04	< 0.001*
Yes	32.87	20.77	

^†^Mann–Whitney, ^‡^Kruskal–Wallis tests *Significant result *p* ≤ 0.05

**Table 6:** The factors associated with high stress (IER-S score): binary logistic regression

	Unadjusted OR				Adjusted OR			
	OR	95% CI		*p* value	OR	95% CI		*p* value
Nationality								
Omani	1.00	–	–	–				
Non-Omani	0.741	0.252	2.178	0.586				
Gender								
Man	1.00	–	–	–	1.00	–	–	–
Woman	1.768	1.156	2.703	0.009	1.895	1.223	2.935	0.004
Place								
Rustaq	1.111	0.339	3.640	0.862				
Musanaah	1.241	0.387	3.980	0.716				
Barka	1.017	0.324	3.197	0.976				
Awabi	0.675	0.121	3.767	0.654				
Wadi Mawel	1.000	0.213	4.693	1.000				
Nakhal	1.246	0.312	4.977	1.246				
Outside SBG	1.00	–	–	–				
Age								
Less than 30	1.00	–	–	–				
31–40	1.124	0.699	1.807	0.628				
More than 40	1.616	0.930	2.809	0.089				
Social status								
Married	0.880	0.325	2.383	0.802				
Single	0.794	0.274	2.300	0.670				
Divorced and widowed	1.00	–	–	–				
Education								
Primary school	1.00							
Secondary school	1.310	0.723	2.373	0.373				
Diploma/bachelor or higher	1.354	0.749	2.449	0.316				
Work								
Government	1.071	0.612	1.877	0.809				
I don’t work	1.207	0.680	2.141	0.520				
Private	1.00	–	–	–				
No of children								
1–2 children	1.086	0.629	1.873	0.768				
More than 3	1.330	0.806	2.193	0.264				
No children	1.00	–	–	–				
No of household members								
5 or less	1.00	–	–	–				
6–10	1.096	0.694	1.731	0.693				
11 or more	0.839	0.456	1.544	0.573				
Comorbidities								
No	1.00	–	–	–				
Yes	1.523	0.926	2.505	0.097				
Duration of isolation								
14 days	1.00	–	–	–				
Less than 14	0.448	0.196	1.024	0.057				
More than 14	1.505	0.967	2.341	0.070				
Regular communication								
No	1.410	0.624	3.182	0.409				
Daily	0.911	0.574	1.447	0.693				
Few times	1.00	–	–	–				
Salary affected								
No	1.00	–	–	–	1.00	–	–	–
Yes	1.845	1.080	3.152	0.025	1.567	0.888	2.765	0.121
Month of diagnosis								
February–June	1.00	–	–	–				
July–November	1.105	0.705	1.732	0.665				
Shortage of essential items								
No	1.00	–	–	–	1.00	–	–	–
Yes	3.071	1.697	5.560	< 0.001	2.928	1.580	5.424	0.001

## Discussion

This cross-sectional study of randomly selected sample in SBG revealed that the prevalence of high stress using two different validated psychological scales K10 and IES_R were 31.9% and 37.7% respectively. Both psychological measures yielded comparable results for associated risk factors. The stress was primarily evident among women and patients with financial difficulties and shortages of essential supplies during the isolation period. Patients experiencing comorbidities and extended isolation periods also had higher stress levels.

Protecting vulnerable patients from stress is imperative. Most participants indicated a willingness to comply with isolation conditions, indicating satisfactory self-awareness. However, a significant minority failed to comply. This failure may have been related to factors such as supply shortages and insufficient awareness.

The Cronbach’s alpha values for K10 and IES-R indicated very good internal consistency. There have been many papers which have suggested different cutoff values for K10’s ability to predict high stress levels. However, we have adopted the less sensitive cutoff value suggested by Andrews and Slade in order to achieve the best estimated prevalence [16]. Conversely, the established cutoff values for the IES-R varied significantly from 22 to 44, which renders its use as a screening tool questionable. Nevertheless, the cutoff value of 24/25 with the specificity (0.75) and sensitivity (0.71) suggested by Nozomu Asukai. et al. is a useful instrument with which to detect survivors of post traumatic distress syndrome (PTDS) [17]. As both measures yielded almost identical results and detected a prevalence rate difference of only 5.8%, this indicates a good reliability and accepted cutoff values.

The isolation protocol requires comprehensive compliance, including separate rooms, masks, remaining inside unless there is a medical emergency and fastidious waste management. However, there were obvious gaps in relation to all variables. This compliance rate, though it appears high, does not eliminate further spread of the disease in the community.

Research from India identified low compliance among children and adolescents[18], while another revealed high compliance (94%) when people were financially compensated, but lower compliance (< 57%) when compensation was removed [9].

In this study, almost 15% of respondents reported supply shortages, which might explain why some patients temporarily broke their confinement.

Previous Canadian research using IES-R revealed 28.9% and 14.6% of respondents experienced stress while quarantined during the 2003 SARS epidemic [4, 19]. Levels reported in the current study were significantly higher. Another study explored the psychological distress experienced by hospital practitioners during the 2015 MERS-CoV outbreak in Korea, where there was a higher mean IES-R (30 + 19.55) among staff who performed MERS tasks, as compared to those in unrelated work (22 + 17.7) [5]. However, the current findings are lower than both these results (19.47 + 7.996). A recently published paper from Australia found a lower prevalence rate of 7.1% for people experiencing quarantine during the COVID-19 pandemic using K10 while the mean score was 13.6 [20].

The current findings accord with research in Turkey that reported that females had OR = 2.478, 95% CI = (1.439, 4.267) for developing anxiety and depression, as compared to men [21]. Previous studies in Oman demonstrated higher levels of stress, anxiety and depression and lower coping scores among females [22, 23]. However, few other studies found gender had no significant impact on psychological distress [24].

The current analysis has revealed that the likelihood of developing high levels of stress was significantly higher among COVID-19 patients when their family income was impacted. In parallel to our findings, a previous study in Oman found income instability to be an independent predictor of psychological distress (OR = 2.05, 95%, CI = 1.54–2.74). In a similar vein, a Chinese study found that family income stability comprises a protective factor against anxiety (OR = 0.726, 95% CI = 0.645–0.817) [22, 25]. Another study in India found that people who had insufficient supplies during lockdown were far more afflicted by anxiety, depression and stress as compared to those who did not experience shortages [26].

The current bivariate analysis with K10 accords with previous research into the psychological impact of the SARS quarantine that found longer durations of quarantine were associated with an increased prevalence of PTSD symptoms [19]. Indeed, coexisting chronic disease and previous psychiatric history were identified as risk factors for health anxiety in a study in Turkey [21].

Stress and other psychiatric problems may continue in the post-pandemic period [27]. Furthermore, psychological disorders also influence the immune system which influences the prognosis for patients with infectious disease [28]. Therefore, policy makers should seek ways to alleviate the stress within the community, not least amongst impacted patients. First, it is necessary to secure all essential supplies required during the isolation period, including food, water, electricity, communication and medical support. Secondly, individuals living under quarantine conditions should receive health exemption in respect of their employment, which should be supported by governmental authority to ensure that it is done properly.

Third, psychiatric counselling and support can be provided through primary health facilities. Fourth, general practitioners must always diagnose or screen for different psychological disorders to identify patients requiring clinical support. Fifth, paramedics and other specialists can use their knowledge and expertise to provide support. The accumulated experience of trained community nurses, for example, could help manage the stress experienced by self-isolating patients through regular visits and follow ups. Sixth, the psychological first aid is often invaluable during stressful situations. Hence, it would be worth training medical and paramedical staff to deliver it. Seventh, implement telemedicine in a health care context could facilitate psychological support. These initiatives can both reduce stress and increase self-isolation compliance. However, additional research is required to determine their precise benefits.

The current study has several limitations. First, in some cases, it was conducted long after the isolation period had been completed. Hence, recall bias is a potential issue. However, the author contends that this effect was reduced by the strong emotions associated with the isolation experience. Moreover, the findings indicate the existence of long-term consequences of self-isolation. Secondly, the study failed to objectively evaluate compliance levels. In addition, it overlooked different risk factors. However, the subjective assessment of compliance levels was persuasive and comparable to the psychological impact. Third, the questionnaire did not undergo advanced validity testing apart from the face and content validity by the expert colleagues. However, the scales used in the study have already been tested in other researches and we also got high reliability score. Therefore, we do assume the scales carried high reliability and validity at the same time. Fourth, the interviews were conducted through introductory phone calls and electronic forms via WhatsApp links, where only we could have verbal consent. Therefore, besides the confidentiality, we ensured participants’ full understanding to the questions in order to avoid any kind of response bias.

## Conclusions

The self-compliance rate is high but not optimal. Moreover, the transmission of infection is possible, especially through the less compliant group. Psychological distress was principally evident among females, participants with reduced income levels and those experiencing supply shortages. In addition, patients with comorbidities and those undergoing extended isolation periods experience more stress, although these factors were not the primary determinants. Interventions are critical to limit stress and enhance compliance with self-isolation restrictions.

## Data Availability

The datasets generated during and/or analysed during the current study are available from the corresponding author on reasonable request.

## Abbreviations

SARS: Severe Acute Respiratory Syndrome
MERS-CoV: Middle East Respiratory Syndrome Coronavirus
K10: Kessler 10 psychological distress
IES-R: The impact of event scale-revised
SBG: South Batinah Governorate
PCR: Polymerase chain reaction
PTDS: Post traumatic distress syndrome
MOH: Ministry of Health
